# Lifetime Upward Economic Mobility and US-Born Latina Women’s Preterm Birth Rates

**DOI:** 10.1007/s10995-023-03890-3

**Published:** 2024-02-03

**Authors:** John Feister, Clarissa Najera, Kristin Rankin, James W Collins

**Affiliations:** 1 Department of Pediatrics, University of Cincinnati College of Medicine, 3230 Eden Avenue, Cincinnati, OH 45267 USA; 2 Division of Neonatal and Pulmonary Biology, Cincinnati Children’s Hospital Medical Center, 3333 Burnet Ave, Cincinnati, OH 45229 USA; 3 School of Public Health, Department of Epidemiology, University of Illinois, Chicago, USA; 4 Division of Neonatology, Ann & Robert H. Lurie Children’s Hospital of Chicago, 225 E. Chicago Ave, Chicago, IL 60611 USA

**Keywords:** Preterm birth, Life-course, Health disparities, Latina paradox

## Abstract

**Objectives:**

To determine whether Latina women’s upward economic mobility from early-life residence in impoverished urban neighborhoods is associated with preterm birth (< 37 weeks, PTB) .

**Methods:**

Multivariate logistic regression analyses were performed on the Illinois transgenerational birth-file with appended US census income information for Hispanic infants (born 1989–1991) and their mothers (born 1956–1976).

**Results:**

In Chicago, modestly impoverished-born Latina women (*n* = 1,674) who experienced upward economic mobility had a PTB rate of 8.5% versus 13.1% for those (*n* = 3,760) with a lifelong residence in modestly impoverished neighborhoods; the unadjusted and adjusted (controlling for age, marital status, adequacy of prenatal care, and cigarette smoking) RR equaled 0.65 (0.47, 0.90) and 0.66 (0.47, 0.93), respectively. Extremely impoverished-born Latina women (*n* = 2,507) who experienced upward economic mobility across their life-course had a PTB rate of 12.7% versus 15.9% for those (*n* = 3,849) who had a lifelong residence in extremely impoverished neighborhoods, the unadjusted and adjusted RR equaled 0.8 (0.63. 1.01) and 0.95 (0.75, 1.22), respectively.

**Conclusions for Practice:**

Latina women’s upward economic mobility from early-life residence in modestly impoverished urban neighborhoods is associated with a decreased risk of PTB. A similar trend is absent among their peers with an early-life residence in extremely impoverished areas.

## Significance

**What is Already Known on this Subject?:**

Robust literature supports a life-course conceptual model of birth outcome. Prior studies have demonstrated associations between upward economic mobility from poverty and decreased rates of preterm birth in non-Latina Black and White women.

**What this Study Adds?:**

Upward economic mobility is associated with decreased rates of preterm birth in US-born Latina women. This finding adds context to the life-course conceptual model of birth outcomes and may be salient to understanding the increased risk of adverse birth outcome among US-born (versus foreign-born) Latina women.

## Introduction

The Hispanic/Latinx population is the second largest and fastest growing ethnic group in the United States, and is projected to nearly double in size in the next 40 years (Colby & Ortman, 2015). US-born Latina women have higher preterm birth (< 37 weeks gestational age) rates than their foreign-born counterparts. This disparity persists regardless of traditional risk factors including race, education attainment, and prenatal care usage (Acevedo-Garcia et al., 2005; Fuentes-Afflick et al., 1998). As such, the birth outcome of US-born Latina women has public health relevance.

Several theories for the observed preterm birth disparity between foreign born and US-born Latina women have been described. The healthy migrant theory posits that immigrants to the US are positively selected for as a function of being healthier than the average population (Rubalcava et al., 2008). The acculturation theory suggests that foreign born women retain healthier cultural influences from their country of origin but adopt increasingly unhealthier behaviors the longer they reside in the US (Scribner & Dwyer, 1989). Finally, the concept of weathering, or the prolonged exposure to inequality, racism, and stress for minorities in the United States, has been investigated for its role in Latina preterm birth (Collins et al., 2012; Powers, 2012; Rich-Edwards & Grizzard, 2005). However, limited research has focused on the importance of changing socioeconomic status over time in the Latina population as an influencer of birth outcome.

Empiric data support a life-course conceptual model of birth outcome in which women’s exposures to contextual level experiences from conception through adulthood are associated with preterm birth (Colen et al., 2006; Collins et al., 2011, 2015; Lu & Halfon, 2003). The conceptual framework proposes major contributions of both early-life (i.e., fetal) imprinting and cumulative stress (i.e. weathering) secondary to low socioeconomic position (Burris & Collins, 2010; Geronimus, 2013; Geronimus et al., 2006). A handful of published investigations have examined the relationship between women’s rising socioeconomic position from early-life to adulthood and preterm birth. A study using the National Longitudinal Survey of Youth found that upward socioeconomic mobility was associated with 50% lower low birth weight (< 2500 g, LBW) rate among non-Latina white women. A similar phenomenon did not occur among African-American women (Colen et al., 2006). However, a separate population-based study found that urban African-American women who experienced upward (compared to no) economic mobility between early-life and adulthood had lower preterm birth rates (Collins et al., 2011). To our knowledge, no study has determined the relationship between US-born Latina women’s upward economic mobility and preterm birth rates.

We, therefore, used the Illinois Transgenerational birth-file with appended US census income information to ascertain the extent to which upward economic mobility is associated with the preterm birth rates of US-born Latina women who had an early-life residence in impoverished Chicago neighborhoods. We hypothesized that impoverished-born urban Latina women who experience upward economic mobility across their life-course have lower preterm birth rates than their counterparts with no upward economic mobility.

## Methods

### Data

This was a retrospective cohort study using data from the Illinois Transgenerational Birth File (TGBF). The creation of the TGBF with appended US census tract income information has been previously described (David et al., 2008). This unique data set that allows for investigation of the association of maternal socioeconomic life course with infant health outcomes. The TGBF includes the computerized birth certificates of infants born to Illinois residents from 1989 to 1991 and their mothers born in Illinois between 1956 and 1976 (*N* = 267,303) and is representative of the Illinois and Chicago-area population.

For the Chicago metropolitan area, US census tract income information is appended to both generations. Census tracts are small regions constructed to follow natural urban boundaries such as highways and railroads, thus approximating real neighborhoods. Thus, neighborhood level income information is available at time of a woman’s birth (early-life) and at the time she gave birth (adulthood).

### Study Sample

The study was restricted to two cohorts: (1) Latina women (*n* = 6,356) with an early-life residence in extremely impoverished (defined by the first quartile of neighborhood income distribution) Chicago neighborhoods and who delivered a singleton live-birth while residing in Chicago or a surrounding Cook County suburb; and (2) Latina women (*n* = 5,434) with an early-life residence in modestly impoverished (defined by the second quartile of neighborhood income distribution) Chicago neighborhoods and who delivered a singleton live-birth while residing in Chicago or the surrounding Cook County suburbs. The median family income of women’s early-life census tract residence ranged from $10,048 to $21,646 in the first quartile and $21,647 to $27,427 (both adjusted for inflation to 1989 USD) in the second quartile.

Covariates included maternal age, marital status, smoking status, and adequacy of prenatal care received (per the Adequacy of Prenatal Care Utilization Index (Kotelchuck, 1994). Infants with missing information on selected covariates were excluded.

### Defining Maternal Upward Economic Mobility

We used the median family income of the woman’s census tract (or the geographically broader community area for the 1956–1960 cohort) at the time they delivered their infants to objectively characterize maternal socioeconomic status at the time of birth. Maternal upward economic mobility was defined by adulthood residence in second, third, or fourth quartile income neighborhoods for the extremely impoverished-born cohort (early-life residence in first quartile of neighborhood income distribution) or adulthood residence in the third or fourth quartile income neighborhoods for the modestly impoverished-born cohort (early-life residence in second quartile of neighborhood income distribution).

The median income of adulthood economic environment for the lowest quartile ranged from $4,999 to $23,425, the second quartile ranged from $23,426 to $35,427, the third quartile ranged from $35,428 to $45,871, and the highest quartile ranged from $45,872 to $150,001 (all in 1989 USD).

### Statistical Analysis

The distribution of women’s age, marital status, adequacy of prenatal care, and cigarette smoking status were determined according to degree of early life impoverishment and upward economic mobility status. To inform later model building, we performed stratum-specific stratified analysis and tested for homogeneity of these stratum-specific RRs (alpha = 0.05) across maternal covariates to identify potential effect modifiers.

Multivariable logistic regression models were created to determine crude and adjusted relative risk (RR & aRR) of preterm birth for extremely and moderately impoverished-born women who experienced upward economic mobility versus no upward economic mobility.

All analyses were performed using SAS software version 9.4 (SAS Institute, Cary NC). The institutional review board of Ann & Robert H. Lurie Children’s Hospital of Chicago approved the research protocol.

## Results

The incidence of maternal age < 20 years, unmarried status, inadequate prenatal care usage, and cigarette smoking was lower among extremely impoverished-born Latina women who experienced upward (compared to no) economic mobility (Table 1). A similar trend occurred among the modestly impoverished-born Latina women who experienced upward (compared to no) economic mobility (Table 2).

**Table 1:** Maternal characteristics for women with and without upward economic mobility from early life extreme impoverishment (i.e. maternal birth in census tract with lowest income quartile)

Maternal Characteristics of Women Born into Extreme Impoverishment		
	No upward economic mobility (*n* = 3849), %	Upward economic mobility (*n* = 2507), %
Age*		
< 20	15	7
20–24	19	16
25–29	38	41
30–35	28	37
Marital Status*		
Unmarried	40	25
Married	60	75
Prenatal Care*		
None/inadequate	24	11
Intermediate	18	13
Adequate	26	46
Adequate+	32	29
Smoking Status*		
Smoker	10	5
Non-Smoker	90	95

*P < .001 for the χ2 test of association between each characteristic and adulthood residential environment. NS = not significant

**Table 2:** Maternal characteristics for women with and without upward economic mobility from early life modest impoverishment (i.e. maternal birth in census tract with second lowest income quartile)

Maternal Characteristics of Women born into Modest Impoverishment		
	No upward economic mobility (*n* = 3760), %	Upward economic mobility (*n* = 1674), %
Age*		
< 20	11	10
20–24	31	17
25–29	41	44
30–35	16	28
Marital Status*		
Unmarried	40	17
Married	60	83
Prenatal Care*		
None/inadequate	19	13
Intermediate	19	17
Adequate	37	57
Adequate+	25	33
Smoking Status^NS^		
Smoker	9	9
Non-Smoker	91	91

*P < .001 for the χ2 test of association between each characteristic and adulthood residential environment. NS = not significant

Table 3 shows the results of multivariable regression models investigating the relationship between upward economic mobility and preterm birth. Impoverished (either extremely or modestly)-born Latina women (*n* = 4,181) who experienced upward economic mobility had a preterm birth rate of 11.0% compared to 14.6% for those (*n* = 7609) with lifelong impoverishment, unadjusted RR = 0.76 (95% CI 0.64–0.82). Extremely impoverished-born Latina women (*n* = 2,507) who experienced upward economic mobility across their life-course had a preterm birth rate of 12.7% compared to 15.9% for those (*n* = 3,849) who had a lifelong residence in extremely impoverished neighborhoods, unadjusted RR = 0.8 (95% CI 0.63–1.01). Modestly impoverished Latina women (*n* = 1,674) who experienced upward economic mobility across their life-course had a preterm birth rate of 8.5% compared to 13.1% for those (*n* = 3,760) with a lifelong residence in modestly impoverished neighborhoods, unadjusted RR = 0.65 (95% CI 0.47–0.90).

**Table 3:** Incidence and relative risk of preterm births (crude and adjusted for maternal age, marital status, prenatal care, smoking status) for women born in varying levels of impoverishment with and without lifetime upward economic mobility

Upward Economic Mobility and Risk of Preterm Birth			
	Preterm Birth Rate (%)	Crude Relative Risk (95% CI)	Adjusted Relative Risk (95% CI)
Any Impoverishment			
No Upward Economic Mobility	14.6	ref	ref
Upward Economic Mobility	11.0	0.76 (0.64–0.90)	0.82 (0.68–0.99)
Extreme Impoverishment			
No Upward Economic Mobility	15.9	ref	ref
Upward Economic Mobility	12.7	0.80 (0.63–1.01)	0.95 (0.75–1.22)
Modest Impoverishment			
No Upward Economic Mobility	13.1	ref	ref
Upward Economic Mobility	8.5	0.65 (0.47–0.90)	0.66 (0.47–0.93)

In multivariate regression models, the adjusted relative risk (controlling for maternal age, marital status, adequacy of prenatal care, and cigarette smoking) of preterm birth for (1) impoverished-born (extremely or modestly) Latina women who experienced any (compared to no) upward economic mobility equaled 0.82 (95% CI 0.68–0.99), (2) extremely impoverished-born Latina women who experienced any (compared to no) upward economic mobility equaled 0.95 (95% CI 0.75–1.22); and (3) modestly impoverished born Latina women who experienced any (compared to no) upward economic mobility equaled 0.66 (95% CI 0.47–0.93).

## Discussion

The present population-based investigation provides new information on the relationship between US-born Latina women’s upward economic mobility and preterm birth rates. Our multilevel, multivariable analyses show that impoverished-born Latina women’s upward economic mobility across their life-course is associated with decreased preterm birth rates, even when adjusting for individual-level risk factors. Interestingly, we find that the degree of early life impoverishment influences the association of upward economic mobility and preterm birth. Extreme impoverishment attenuates the association between upward economic mobility and preterm birth. In contrast, we find that modestly impoverished-born Latina women’s upward economic mobility is associated with lower preterm birth rates independent of age, marital status, prenatal care usage and cigarette smoking. These findings are salient to the known phenomenon of US-born (compared to foreign-born) Latina women’s increased risk of preterm birth.

Our data show that modestly impoverished-born Latina women’s upward (compared to no) economic mobility is associated with substantially lower preterm birth regardless of well-known individual-level risk factors. This phenomenon is consistent with an underlying contextual process closely related to modestly impoverished-born upwardly mobile Latina women’s improved lifetime exposure to health promoting environments. Potential mechanisms include decreased lifetime exposure to the effects of structural racism and extreme poverty, including lower levels of neighborhood violence (Masho et al., 2017; Messer et al., 2006), diminished contact with environmental toxins (Klepac et al., 2018; Trasande et al., 2016), and improved access to high quality preventative health care services (Cox et al., 2011; Vintzileos et al., 2002). Further studies should focus on potential causal mechanisms of upward economic mobility on improvement in birth outcomes.

We find no statistically significant reduction in risk of preterm birth for upwardly economic mobile women born in extreme impoverishment. This finding suggests upward economic mobility alone may not be sufficient to overcome the severity of the effects of early life extreme impoverishment on future maternal health. Alternatively, there may be other unmeasured characteristics associated with early life in extreme impoverishment that influence risk of preterm birth.

Our data highlight an elevated preterm birth rate among US-born Latina women with an early life and adulthood residence in impoverished areas. The overall preterm birth rate in 1990 for women born in the US was 10.7% (11.0% among Latina women) (National Center for Health Statistics, 1994). Our finding of a markedly higher preterm birth rate of 14.6% among US-born Latina women with lifelong residence in impoverishment demonstrates the importance of considering lifelong socioeconomic status in large scale population studies of natality and must be considered when considering the paradox of supposedly better than expected birth outcomes for the Latina population. Few published studies have examined the relationship between residential environment and preterm birth rates among Latina women giving birth in the US. A prior population-based study found that adulthood residence in low-income urban neighborhoods was associated with low birth weight rates among US-born but not foreign-born Latina women (Collins & Shay, 1994), suggesting a causal relationship between the former’s lifelong exposure to neighborhood poverty and an increased risk of preterm birth. Alternatively, foreign-born Latina women’s lower preterm birth rate may reflect their shorter duration of exposure to factors closely related to underserved minority status, particularly neighborhood impoverishment.

Notwithstanding, our data provide a plausible contextual explanation for US-born (compared to foreign-born) Latina women’s increased risk of delivering preterm birth infants. A significant percentage of urban US-born Latina women have early-life residence in impoverished neighborhoods, with 28% of all Hispanic children living in poverty (U.S. Census Bureau, 2016). The long lasting effects of adverse childhood experiences associated with poverty are well documented in the medical literature (Hughes et al., 2017; Walsh et al., 2019). We speculate that this observation of elevated risk of preterm birth in life long impoverished women reflects the lingering impact of US-born women’s unmet heath needs during early-life and childhood (Halfon et al., 2017; Larson & Halfon, 2010). We encourage researchers to take neighborhood income across the life-course into account when examining the birth outcome of US-born Latina women.

Our findings are of particular importance to policy makers. Policies that promote upward economic mobility, such as investment in social safety net programs, community infrastructure, education, and programs that support familial wealth-building may have multiplier effects by reducing costly preterm birth in already vulnerable populations. We encourage policy makers to consider preterm birth and its associated costs when evaluating the impact of these policies.

The Illinois TGBF is a rare population-based vital record dataset of US births with appended US census income information; however, it has pertinent limitations (Collins et al., 2011; David et al., 2008). First, due to sample size considerations Latina women categorized into a single category. In Chicago, Mexican-Americans are the largest Hispanic/Latinx subgroup followed by Puerto Ricans. Our findings may not be generalizable to urban cities with different distributions of Latinx subgroups. Additionally, we were unable to capture Chicago-born women who migrated out of the Chicago metropolitan area. Another potential limitation is that the preterm birth rates from 1989 to 1991 may not be generalizable to contemporary births. Lastly, we did not adjust for parity, which is known to influence risk of preterm birth, in this study.

In summary, this study demonstrates that lifetime upward economic mobility in Chicago-born Latina women is associated with decreased rates of preterm birth. Extreme impoverishment appears to have a threshold at which upward economic mobility is not associated with statistically significant reduction in preterm birth. This study contributes to the body of literature supporting a life-course conceptual model of birth outcomes and adds understanding to the “Latina paradox” in birth outcomes.

## Data Availability

Upon request.

## Code Availability

Upon request. SAS.
